# Computational Modeling-Based Discovery of Novel Classes of Anti-Inflammatory Drugs That Target Lanthionine Synthetase C-Like Protein 2

**DOI:** 10.1371/journal.pone.0034643

**Published:** 2012-04-11

**Authors:** Pinyi Lu, Raquel Hontecillas, William T. Horne, Adria Carbo, Monica Viladomiu, Mireia Pedragosa, David R. Bevan, Stephanie N. Lewis, Josep Bassaganya-Riera

**Affiliations:** 1 Center for Modeling Immunity to Enteric Pathogens, Virginia Tech, Blacksburg, Virginia, United States of America; 2 Nutritional Immunology and Molecular Medicine Laboratory, Virginia Bioinformatics Institute, Virginia Tech, Blacksburg, Virginia, United States of America; 3 Department of Biochemistry, Virginia Tech, Blacksburg, Virginia, United States of America; Bioinformatics Institute, Singapore

## Abstract

**Background:**

Lanthionine synthetase component C-like protein 2 (LANCL2) is a member of the eukaryotic lanthionine synthetase component C-Like protein family involved in signal transduction and insulin sensitization. Recently, LANCL2 is a target for the binding and signaling of abscisic acid (ABA), a plant hormone with anti-diabetic and anti-inflammatory effects.

**Methodology/Principal Findings:**

The goal of this study was to determine the role of LANCL2 as a potential therapeutic target for developing novel drugs and nutraceuticals against inflammatory diseases. Previously, we performed homology modeling to construct a three-dimensional structure of LANCL2 using the crystal structure of lanthionine synthetase component C-like protein 1 (LANCL1) as a template. Using this model, structure-based virtual screening was performed using compounds from NCI (National Cancer Institute) Diversity Set II, ChemBridge, ZINC natural products, and FDA-approved drugs databases. Several potential ligands were identified using molecular docking. In order to validate the anti-inflammatory efficacy of the top ranked compound (NSC61610) in the NCI Diversity Set II, a series of *in vitro* and pre-clinical efficacy studies were performed using a mouse model of dextran sodium sulfate (DSS)-induced colitis. Our findings showed that the lead compound, NSC61610, activated peroxisome proliferator-activated receptor gamma in a LANCL2- and adenylate cyclase/cAMP dependent manner *in vitro* and ameliorated experimental colitis by down-modulating colonic inflammatory gene expression and favoring regulatory T cell responses.

**Conclusions/Significance:**

LANCL2 is a novel therapeutic target for inflammatory diseases. High-throughput, structure-based virtual screening is an effective computational-based drug design method for discovering anti-inflammatory LANCL2-based drug candidates.

## Introduction

Prokaryotic LanC is a part of a multimeric membrane-associated lanthionine synthetase complex involved in the modification and transport of peptides. LanC itself is a zinc-containing enzyme that acts in concert with specific dehydratases to facilitate intramolecular conjugation of cysteine to serine or threonine residues, yielding macrocyclic thioether analogs of cysteine known as lanthionines. These products display potent antimicrobial activity, and are also known as lantibiotics [1]. The first member of the eukaryotic lanthionine synthetase component C-like (LANCL) protein family, LANCL1, was isolated from human erythrocyte membranes [2]. A related protein, LANCL2, was subsequently identified in human brain and testis [3]. LANCL1 and 2 have similar expression patterns, with strong expression in brain and testis, and weak but ubiquitous expression in other tissues [2], [3]. LANCL2 is most highly expressed in testis, and its exogenous introduction has been shown to cause increased cellular sensitivity to the anticancer drug, adriamycin, by suppressing the expression of MultiDrug-Resistance 1 and its cognate protein, P-glycoprotein [4]. On the other hand, overexpressed LANCL2 interacted with the actin cytoskeleton, implying that LANCL2 may also have a role in cytoskeletal reorganization and cellular movement [5].

Sturla and colleagues provided *in vitro* results suggesting that LANCL2 is required for abscisic acid (ABA) binding to the membrane of human granulocytes and for transduction of the ABA signal into cell-specific functional responses in granulocytes [6]. ABA is an isoprenoid phytohormone that plays important roles in plant responses to environmental stresses and host responses [7]. In addition, ABA has received recent attention due its peroxisome proliferator-activated receptor (PPAR) γ-activating and anti-inflammatory properties, which make it a target for development of potent anti-inflammatory and insulin-sensitizing therapeutics [7]. We demonstrated that PPAR γ is required for ABA to induce its full spectrum of effects, but ABA does not bind directly to the ligand-binding domain (LBD) of PPAR γ [8]. The mechanism of activation of PPAR γ by ABA is not completely understood, but there is evidence supporting the observation that ABA-mediated PPAR γ activation requires expression of LANCL2 in immune cells [8]. Indeed, we demonstrated that ABA binds to LANCL2 *in silico* [8]. Moreover, by using molecular modeling approaches, we elucidated the location of the potential LBD of LANCL2 for ABA. Recently, a series of *in vitro* binding studies on human LANCL2 recombinant protein confirmed direct binding of ABA to LANCL2, including saturation binding, scintillation proximity assays, dot blot experiments, and affinity chromatography [9]. Identification of ABA binding to LANCL2 paves the way for the discovery and development novel anti-inflammatory drugs that target LANCL2. Based on previous findings, we proposed that LANCL2 might be a putative novel target for the discovery and development of orally active, broad-based drugs against inflammatory, infectious and chronic metabolic diseases [10].

The predominant technique employed in the identification of new drugs is the physical large scale, high-throughput screening of thematic compound libraries against a biological target, which is very costly and yields mixed results. Recent successes in predicting new ligands and their receptor-bound structures make use of structure-based virtual screening (SBVS), which is a more cost-effective approach in drug and nutraceutical discovery. The basic procedure of SBVS is to sample binding geometry for compounds from large libraries into the structure of receptor targets by using molecular modeling approaches. Each compound is sampled in thousands to millions of possible poses and scored on the basis of its complementarity to the receptor. Of the hundreds of thousands of molecules in the library, tens of top-scoring predicted ligands are subsequently tested for activity in experimental assays [11]. One of the main requirements for SBVS is availability of the three-dimensional structure of a validated protein target [12]. In some cases, when the crystal structure of the receptor target is unknown, computer-modeled structures have been verified to suffice for successful virtual screening [13], [14], [15], [16]. In a previous study from our group, homology modeling of human LANCL2 was performed using the crystal structure of human LANCL1 as a template [17] and the model quality was assessed [10].

We performed LANCL2-based virtual screening using the structure of LANCL2 obtained through homology modeling to discover new LANCL2 agonists. Thousands of compounds from NCI Diversity Set II, ChemBridge, ZINC natural products and U.S. Food and Drug Administration (FDA)-approved drug databases were docked into the LANCL2 model and ranked by the calculated affinity. The effect of the top ranked compound in the NCI Diversity Set II, the benzimidazophenyl compound denoted NSC61610, on the activity of PPAR γ was tested *in vitro* using a dual luciferase reporter activity assay. Its *in vivo* efficacy and cell-specific PPAR γ dependency were then examined using a mouse model of experimental IBD.

## Materials and Methods

### Expression of LANCL2 in Mouse Tissues

Proteins were extracted from different mouse tissue, including thymus, lung, spleen, stomach, ileum, colon, Peyer's patches (PP), mesenteric lymph node (MLN), gastric lymph node (GLN), blood, white adipose tissue (WAT), and bone marrow (BM) as previously described [8]. The protein extracts were analyzed using a 10% SDS-PAGE gel in a Bio-Rad mini-gel box running condition (75 V×3 hrs). Afterward, proteins were electrotransferred to nitrocellulose by standard methods along with the Precision Plus Kaleidoscope Standard (BIO-RAD). Filters were blocked by 5% BSA in TBS-Tween for 1 hour, followed by incubation with rabbit anti-LANCL2 primary antibody (SIGMA-ALDRICH) in TBS-Tween for 6 hours at room temperature. Goat anti-rabbit horseradish peroxidase-conjugated secondary antibody (Santa Cruz Biotechnology) was used at a dilution of 1∶2000 in TBS-Tween, and protein bands were detected with Immun-StarTM chemiluminescent substrate (BIO-RAD). Re-probing western blot was applied by incubating nitrocellulose in stripping buffer (Thermo scientific) for 15 minutes.

### Compound Database Management and Ligand Structure

The structure files of compounds were obtained from the ZINC database in mol2 format [18], and the individual mol2 files were converted into pdbqt files using the python script prepare_ligand4.py available in the Autodock Tools package [19]. The NCI diversity set II is a reduced set of 1,364 compounds selected from the almost 140,000 compounds available for distribution from the DTP (Developmental Therapeutics Program) repository. The selection process is outlined in more detail at the NCI DTP website (http://www.dtp.nci.nih.gov/branches/dscb/div2_explanation.html). The ChemBridge Corporation maintains a stock of more than 800,000 drug-like and lead-like screening compounds. Structures for these compounds are available for download from the ZINC database (http://zinc.docking.org/vendor0/chbr/index.html). The ZINC natural products database has a structure collection of 89,425 natural products available for download from the ZINC database (http://zinc.docking.org/vendor0/npd/index.html). The FDA-approved drugs database includes 3,180 FDA-approved drug structures, which also are available for download from the ZINC database (http://zinc.docking.org/vendor0/fda/index.html).

### Virtual Screening

The docking of compounds available in the NCI Diversity Set II, ChemBridge, ZINC natural products and FDA-approved drugs databases into the LANCL2 computational model was performed with AutoDock Vina (version 1.0) [20]. AutoDockTools, the graphical front-end for AutoDock and AutoGrid, was used to define the search space, including grid box center and x,y,z-dimensions [19]. A variety of stochastic global optimization approaches were used in AutoDock Vina, including genetic algorithms, particle swarm optimization, simulated annealing and others. Five bound conformations were generated by AutoDock Vina for each compound. The docking was applied to the whole protein target, with a grid covering the whole surface of the protein. To search the entire surface of LANCL2, grid maps were set with the maximum spacing between grid points. The grid was a rectangular cuboid (70 Å×70 Å×60 Å) with grid points separated by 1.000 Å and centered at the middle of the protein. This grid was big enough to cover the entire surface of LANCL2.

### Analyzing the Virtual Screening Results

The search for the best way to fit each compound into LANCL2 using AutoDock Vina resulted in docking log files that contained records of docking, including binding energy of each predicted binding mode for all the compounds. Binding energies represent the sum of the total intermolecular energy, total internal energy and torsional free energy minus the energy of the unbound system. For each database, compounds were ranked by the most negative energy value. All predicted binding poses were placed into one multimodel PDBQT file.

### PPAR γ Reporter Activity Assays on Raw Macrophages

To determine PPAR γ activity, pCMX.PPAR γ expression plasmid and a pTK.PPRE3x luciferase reporter plasmid driven by the peroxisome proliferator responsive element-containing Acyl-CoA oxidase promoter were purified using maxi kit from Qiagen (Valencia, CA). RAW 264.7 macrophages were cultured with DMEM (Mediatech, Manassas, VA) containing 10% fetal bovine serum (FBS) and grown until 60–70% confluence. Cells were cotransfected in two 25 cm2 flasks with 1.5 µg plasmid of DNA and 0.15 µg of pRL reporter control with or without 100 pmol LANCL2 siRNA using Lipofectamine 2000 transfection reagent (Invitrogen) according to the manufacturer's protocol. After 24 h, transfected cells were seeded into white, opaque 96-well plates (BD Biosciences) at a concentration of 25,000 cells/well. Transfected cells were then treated in replicates with rosiglitazone (Ros 1 µM; Cayman Chemical, Ann Arbor, MI), NSC61610 (2.5 µM) with and without 2′5′-dideoxyadenosine (10 µM; Sigma) or vehicle (DMSO) and placed in a 37°C incubator with 5% CO2. After 20 h, cells were harvested in reporter lysis reagent. Luciferase activity, normalized to pRL activity in the cell extracts, was determined by using the Dual-Luciferase II reporter assay system (Promega, Madison, WI) using a Modulus 96-well luminometer (Turner Biosystems, Sunnyvale, CA). All values were normalized to control wells to calculate relative luciferase activity.

### Ethics Statement

All experimental procedures were approved by the Institutional Animal Care and Use Committee (IACUC) of Virginia Tech and met or exceeded requirements of the Public Health Service/National Institutes of Health and the Animal Welfare Act. The IACUC approval ID for the study was 11-057-VBI.

### Animal Procedures to Test the Anti-inflammatory Efficacy of Lead Compound NSC61610 in IBD

Eight week old C57BL/6J mice were housed at the animal facilities at Virginia Tech in a room maintained at 75°F, with a 12:12 hr light-dark cycle starting from 6:00 AM. Mice were randomly assigned into four groups: a control group including 8 mice and the other three NSC61610 treatment groups containing 10 mice each. The three treatment groups received 0.5, 10 or 20 mg NSC61610/kg body weight by orogastric gavage for 7 days. All the mice were challenged with 2.5% DSS, 36,000–44,000 molecular weight (ICN Biomedicals, Aurora, OH) in the drinking water for 7 days. Mice were weighed on a daily basis and examined for clinical signs of disease associated with colitis (i.e., perianal soiling, rectal bleeding, diarrhea, and piloerection). For the DSS challenge, the disease activity indices (DAIs) and rectal bleeding scores were calculated using a modification of a previously published compounded clinical score [21]. Briefly, DAI consisted of a scoring for diarrhea and lethargy (0–3), whereas rectal bleeding consisted of a visual observation of blood in feces and the perianal area (0–4). On day 7 of the challenge, mice in the DSS study were euthanized.by CO_2_ narcosis followed by secondary thoracotomy and blood was drawn from the heart. Colon, spleen, and MLN were scored based on size and macroscopic inflammatory lesions (0–3), excised, and single-cell suspensions were prepared for flow cytometric analyses.

### Histopathology

Colonic sections were fixed in 10% buffered neutral formalin, later embedded in paraffin, and then sectioned (5 mm) and stained with H&E stain for histologic examination. Colons were blindly graded with a compounded histologic score including the extent of (1) leukocyte infiltration, (2) mucosal thickening, and (3) epithelial cell erosion. The sections were graded with a score of 0–4 for each of the previous categories and data were analyzed as a normalized compounded score as previously described [21].

### Quantitative Real-time RT-PCR

Total RNA was isolated from colons using the RNA isolation Minikit (Qiagen) according to the manufacturer's instructions. Total RNA (1 mg) was used to generate complementary DNA (cDNA) template using the iScript cDNA Synthesis Kit (Bio-Rad, Hercules, CA). The total reaction volume was 20 µL with the reaction incubated as follows in an MJ MiniCycler: 5 min at 25°C, 30 min at 52°C, 5 min at 85°C, and hold at 4°C. PCR was performed on the cDNA using Taq DNA polymerase (Invitrogen, Carlsbad, CA) and using previously described conditions. Each gene amplicon was purified with the MiniElute PCR Purification Kit (Qiagen) and quantitated on an agarose gel by using a DNA mass ladder (Promega). These purified amplicons were used to optimize quantitative real-time RT-PCR conditions and to generate standard curves. Primer concentrations and annealing temperatures were optimized for the iCycler iQ system (Bio-Rad) for each set of primers using the system's gradient protocol. PCR efficiencies were maintained between 92 and 105% and correlation coefficients above 0.98 for each primer set during optimization and also during the real-time PCR of sample DNA.

cDNA concentrations for genes of interest were examined by RT-PCR using an iCycler IQ System and the iQ SYBR green supermix (Bio-Rad). A standard curve was generated for each gene using 10-fold dilutions of purified amplicons starting at 5 pg of cDNA and used later to calculate the starting amount of target cDNA in the unknown samples. SYBR green I is a general double-stranded DNA intercalating dye and may therefore detect non-specific products and primer/dimers in addition to the amplicon of interest. In order to determine the number of products synthesized during the real-time PCR, a melting curve analysis was performed on each product. RT- PCR was used to measure the starting amount of nucleic acid of each unknown sample of cDNA on the same 96-well plate. Results are presented as starting quantity of target cDNA (picograms) per microgram of total RNA as previously described [21]. Primer sequences and Genebank accession numbers are outlined in Table S1.

### Immunophenotyping of tissues of mice with IBD

Colonic lamina proprial lymphocytes (LPL) were isolated from digested colons. Spleens and MLNs were excised and single cell suspensions were prepared. Splenocytes were freed of red blood cells with erythrocyte lysis buffer, and spleen and MLN were resuspended in PBS and enumerated by using a Coulter Counter (Beckman Coulter, Fullerton, CA). LPL, spleen and MLN-derived cells (2×10^5^ cells/well) or whole blood (10 µL/well) were seeded onto 96-well plates, centrifuged at 4°C at 3000 rpm for 4 min, and washed with PBS containing 5% serum and 0.09% sodium azide (FACS buffer). To assess differential monocyte/macrophage infiltration, the cells were then incubated in the dark at 4°C for 20 min in FcBlock (20 µg/ml, BD Pharmingen) for macrophage assessment, and then for an additional 20 min with fluorochrome-conjugated primary antibodies anti-F4/80-PE-Cy5 (0.2 mg/mL, ebioscience) and anti-CD11b-Alexa Fluor 700 (0.2 mg/mL, BD Pharmingen). For lymphocyte subset assessment, cells were incubated with anti-CD45-APC-Cy7 (for LPL only) (0.2 mg/mL, BD Pharmingen), anti-CD4-PE-Cy7 (0.2 mg/mL, BD Pharmingen), anti-CD8-PerCp-Cy5.5 (0.2 mg/mL, eBioscience), anti-CD3-PE-Cy5 (0.2 mg/mL, ebioscience), anti-FoxP3-APC (0.2 mg/mL, eBioscience), and anti-IL10-FITC (0.5 mg/mL, BD Pharmingen). Flow results were computed with a BD LSR II flow cytometer and data analyses were performed with FACS Diva software (BD).

### Characterization of the Immunoregulatory Mechanisms of NSC61610 in Mice with Experimental IBD

PPAR γ fl/fl Cre- (n = 20), tissue-specific PPAR γ fl/fl CD4-Cre+ (T cell-deficient) PPAR γ null mice (n = 20) and tissue-specific PPAR γ fl/fl Lysozyme M-Cre+ (myeloid-deficient) PPAR γ null mice (n = 20) in a C57BL/6J background were generated by using the Cre-lox recombination system as previously described [21]. In each group, 20 mice were randomly divided into two groups: a control group including 10 mice and a NSC61610 treatment group containing 10 mice. The three treatment groups received 20 mg/kg NSC61610 by orogastric gavage for 6 days. We selected 20 mg/kg for subsequent testing in this study because this dose had shown the greatest anti-inflammatory activity in the dose-response study (described above). All the mice (n = 60) were challenged with drinking water containing 2.5% DSS, 36,000–44,000 molecular weight (ICN Biomedicals, Aurora, OH) for 6 days. Mice were weighed on a daily basis and examined for clinical signs of disease associated with colitis. For the DSS challenge, the disease activity indices and rectal bleeding scores were calculated using a modification of a previously published compounded clinical score [21]. Mice in the DSS study were euthanized on day 6 of the DSS challenge. On day 6, colon, spleen, and MLN were scored based on size and macroscopic inflammatory lesions (0–3), excised, and then crushed to produce single-cell suspensions for flow cytometry.

### Statistics

Data were analyzed as a completely randomized design. To determine the statistical significance of the model, analysis of variance (ANOVA) was performed using the general linear model procedure of Statistical Analysis Software (SAS), and probability value (*P*)<0.05 was considered to be significant. When the model was significant, ANOVA was followed by multiple comparison method to identify pairwise treatments with significant difference.

### Reverse docking NSC61610 to Novel Potential Drug Targets

The potential drug target database (PDTD) is a dual function database that associates an informatics database to a structural database of known and potential drug targets. PDTD is a comprehensive, web-accessible database of drug targets, and focuses on those drug targets with known 3D-structures. The target proteins collected in PDTD were selected from the literature, and from several online databases, such as DrugBank and Therapeutic Targets Database (TTD). PDTD contains 1,207 entries covering 841 known and potential drug targets with structures from the PDB. Drug targets of PDTD were categorized into 15 and 13 types, respectively, according to two criteria: therapeutic areas and biochemical criteria [22]. Target Fishing Dock (TarFisDock) is a web-based tool for seeking potential binding proteins for a given ligand. It applies a ligand-protein reverse docking strategy to search out all possible binding proteins for a small molecule from the PDTD [23]. TarFisDock was developed on the basis of DOCK (version 4.0) program [24]. The reverse docking procedure is as follows: 1) The NSC61610 structure file in sdf format was downloaded from PubChem (SID 109036). The NSC61610 structure file was transformed to the standard mol2 format using the Chimera program [25]. 2) TarFisDock docked NSC61610 into the possible binding sites of proteins in the target list. Putative binding proteins are selected by ranking the values of the interaction energy, which is composed of van der Waals and electrostatic interaction terms.

## Results

### Virtual Screening and Result Analysis

**Figure 1:**
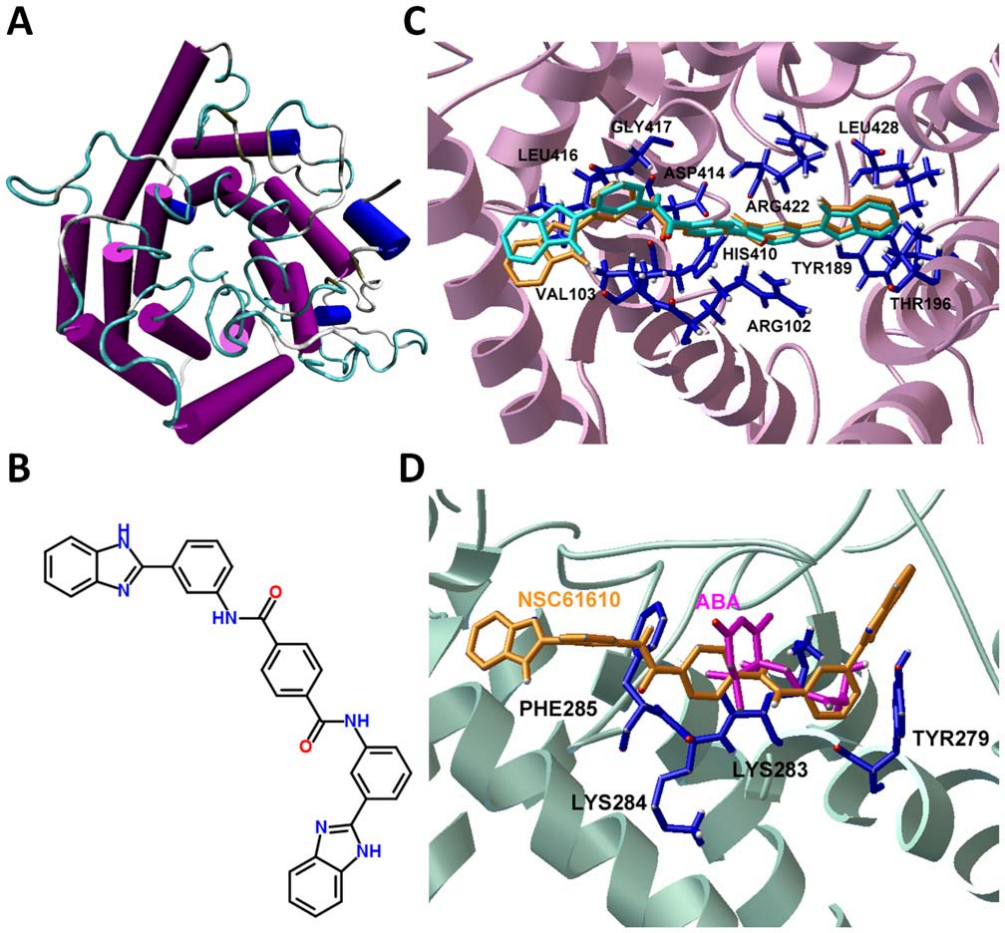
Lanthionine synthetase C-like 2 (LANCL2) and NSC61610 *in silico*. (A) The homology model of human LANCL2 is shown in Cartoon representation with coloring according to secondary structure. Purple: alpha helix; Blue: other helix; Yellow: bridge_beta; Cyan: turn; Green: coil. (B) 2-D structure of NSC61610. (C) Representative binding mode of the most stable docked orientation of NSC61610 with LANCL2. The LANCL2 model is shown in ribbon mode. NSC61610 pose generated by AutoDock Vina is colored in cyan and the one generated by AutoDock is colored in orange. Selected residues of LANCL2 (blue) are depicted by stick-and-ball models and colored by atom types. Amino acid residues surrounding NSC61610 are labeled. (D) Representative binding modes of the docked orientation of abscisic acid (ABA) and NSC61610 with LANCL2. LANCL2 is shown in a ribbon mode. NSC61610 (orange) and ABA (magenta) are shown in stick-and-ball model. Selected residues of LANCL2 surrounding both NSC61610 and ABA are depicted by stick-and-ball model and labeled. The images were rendered in Visual Molecular Dynamics (VMD).

In a previous study, we constructed the homology model of LANCL2 according to the crystal structure of LANCL1 using SWISS-MODEL Workspace [26]. The structure of LANCL2 is shown in Figure 1A [27]. Two levels of assessment, ANOLEA [28] and PROCHECK [29], both indicated the good quality of the model [10].

To discover novel naturally occurring compounds, new drugs and repurposed drugs that target the LANCL2 and potentially exert insulin-sensitizing and anti-inflammatory actions, virtual screening was applied to identify potential ligands of LANCL2. The compound databases used for screening contain NCI Diversity Set II, ChemBridge and ZINC natural product, existing drug databases and FDA-approved drugs databases.

During the *in silico* screening process, compounds were ranked according to their estimated free energy of binding. The best ten docking solutions based on the energy scores were selected for each database (Tables S2, S3, S4, and S5) using AutoDock Vina. A lower binding free energy indicates a more stable protein-ligand system and a higher affinity between protein and ligand. In our integrated discovery pipeline, lead compounds in each category are further validated by *in vitro* testing and pre-clinical studies using mouse models of human diseases. NSC61610, the structure of which is given in Figure 1B, had the lowest free energy of binding (−11.1 kcal/mol) compared to other compounds in NCI Diversity Set II. To further verify the binding between NSC61610 and LANCL2 *in silico*, we docked NSC61610 to LANCL2 using AutoDock (version 4.2) [19]. The detailed approach is the same as the one we applied in docking experiments of ABA [10]. The 100 resulting poses of NSC61610 were clustered with an RMSD cluster tolerance of 2.0 Å. The lowest binding energy pose in the first cluster was considered as the most favorable docking pose. The region on the LANCL2 with the first cluster was considered as the potential binding site for NSC61610. By comparing the amino acid residues involved in binding sites, we found the binding sites predicted by the two different docking programs were identical to each other in most regions. The shared amino acid residues included ARG102, VAL103, TYR189, THR196, ASP414, LEU416, GLY417, ARG422, and LEU428 (Fig. 1C). Thus, the identical region on the LANCL2 identified by the two docking programs was considered as the potential binding site for NSC61610. In addition to this binding site, AutoDock also identified a binding site for NSC61610 on LANCL2 that is similar to the binding site for ABA (Fig. 1D) [10]. Since the binding energy is only one of many possible criteria for identifying potential binding sites, further experimentation was needed to verify the binding site of NSC61610 or determine whether NSC61610 has multiple binding sites on LANCL2.

### Knockdown of LANCL2 Disrupts NSC61610-induced PPAR γ Activation

**Figure 2:**
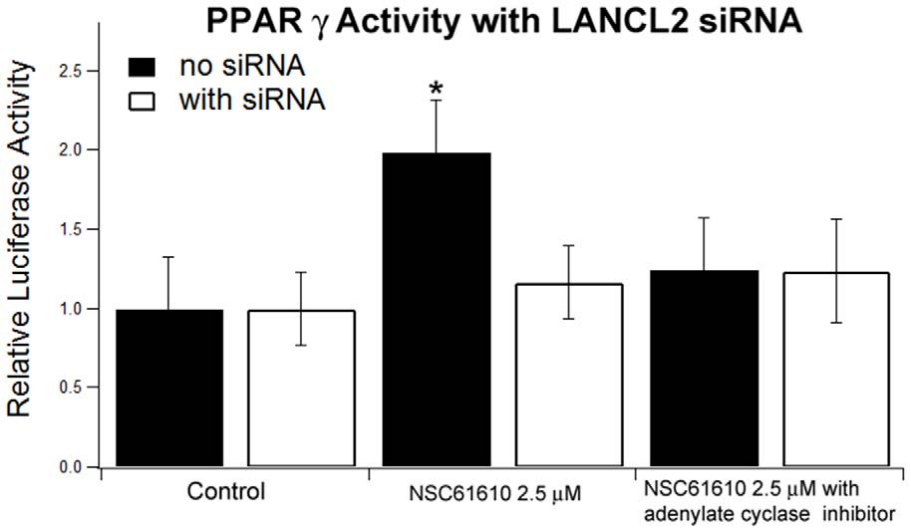
Effect of LANCL2 disruption and cAMP inhibition on PPAR γ activation in RAW 264.7 macrophages. Cells were cotransfected with a pTK.PPRE3x luciferase reporter plasmid driven by the PPRE-containing Acyl-CoA oxidase promoter with or without LANCL2 siRNA. Then, cells were treated with vehicle (DMSO) or NSC61610 (2.5 µM), the adenylate cyclase-specific inhibitor 2′5′-dideoxyadenosine (10 µM). Luciferase activity was normalized to pRL activity in the cell extracts and relative luciferase activity was calculated a ratio of the activity in the treatment wells to control wells. Data are represented as mean ± standard error. Points with an asterisk indicate that a treatment is significantly different from its control (*P<0.05*).

To determine PPAR γ activity, pCMX.PPAR γ expression plasmid and a pTK.PPRE3x luciferase reporter plasmid driven by the peroxisome proliferator responsive element-containing Acyl-CoA oxidase promoter were purified using maxi kit from Qiagen (Valencia, CA). RAW 264.7 macrophages were cultured with DMEM (Mediatech, Manassas, VA) containing 10% fetal bovine serum (FBS) and grown until 60–70% confluence. Cells were cotransfected in two 25 cm2 flasks with 1.5 µg plasmid of DNA and 0.15 µg of pRL reporter control with or without 100 pmol LANCL2 siRNA using Lipofectamine 2000 transfection reagent (Invitrogen) according to the manufacturer's protocol. After 24 h, transfected cells were seeded into white, opaque 96-well plates (BD Biosciences) at a concentration of 25,000 cells/well. Transfected cells were then treated in replicates with rosiglitazone (Ros 1 µM; Cayman Chemical, Ann Arbor, MI), NSC61610 (2.5 µM) with and without 2′5′-dideoxyadenosine (10 µM; Sigma) or vehicle (DMSO) and placed in a 37°C incubator with 5% CO2. After 20 h, cells were harvested in reporter lysis reagent. Luciferase activity, normalized to pRL activity in the cell extracts, was determined by using the Dual-Luciferase II reporter assay system (Promega, Madison, WI) using a Modulus 96-well luminometer (Turner Biosystems, Sunnyvale, CA). All values were normalized to control wells to calculate relative luciferase activity. In the same project, we assessed whether introduction of LANCL2 siRNA affects NSC61610-induced PPAR γ activation. To measure the effect of LANCL2 knockdown on NSC61610-induced PPAR γ activation, raw macrophages were transfected with a PPAR γ expression and dual luciferase plasmids with or without l LANCL2 siRNA, and treated with NSC61610 (2.5 µM). Our data indicate that the addition of LANCL2 siRNA disrupted PPAR γ activation by NSC61610 (Fig. 2).

### NSC61610 activates PPAR γ by AC-cAMP Signaling Pathway

To determine whether NSC61610-induced activation of PPAR γ is dependent on adenylate cyclase (AC)-cyclic adenosine monophosphate (cAMP) signaling, raw macrophage were treated with NSC61610 (2.5 µM) with or without 2′5′-dideoxyadenosine (10 µM). NSC61610 increased PPAR γ activity and addition of the AC-specific inhibitor prevented NSC61610-induced PPAR γ activation (Fig. 2). We also examined whether the inhibitor influenced PPAR γ activity in raw macrophage with knockdown of LANCL2. The PPAR γ activity was not further reduced by addition of AC-specific inhibitor (Fig. 2).

### NSC61610 Ameliorates Disease Activity and Inflammatory Lesions in Mice with IBD

**Figure 3:**
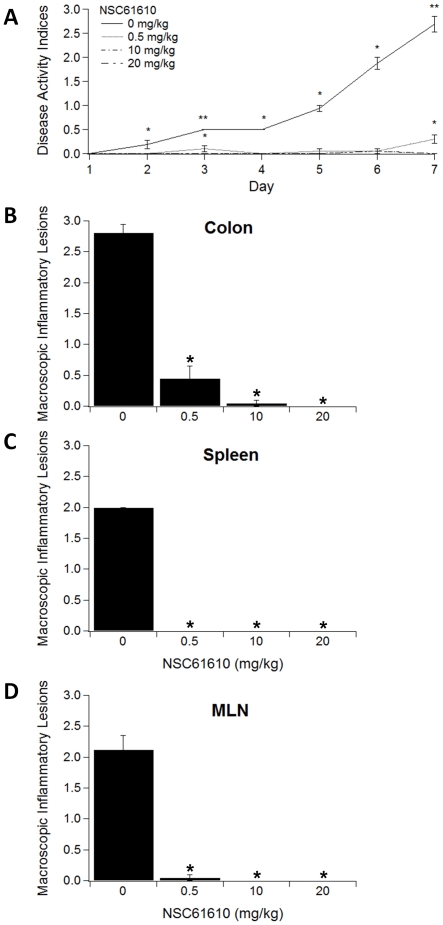
Oral treatment with NSC61610 ameliorates experimental inflammatory bowel disease. Mice were challenged with 2.5% dextran sodium sulfate in the drinking water for 7 days. Disease activity index (DAI), a composite score reflecting clinical signs of the disease (i.e. perianal soiling, rectal bleeding, diarrhea and piloerection), was assessed daily. Panel A illustrates the effect of NSC61610 on disease severity in mice with colitis. Panels B–D illustrate the effect of NSC61610 on macroscopic inflammatory lesions in the colon (B), spleen (C), and mesenteric lymph nodes (MLN) (D). Data are represented as mean ± standard error (n = 10). In figure A, data points with asterisks are significantly different from control and data points with two asterisks are significantly different from those with one asterisk (*P<0.05*). In figure B–D, bars with an asterisk indicate that a treatment is significantly different from its control (*P<0.05*).

**Figure 4:**
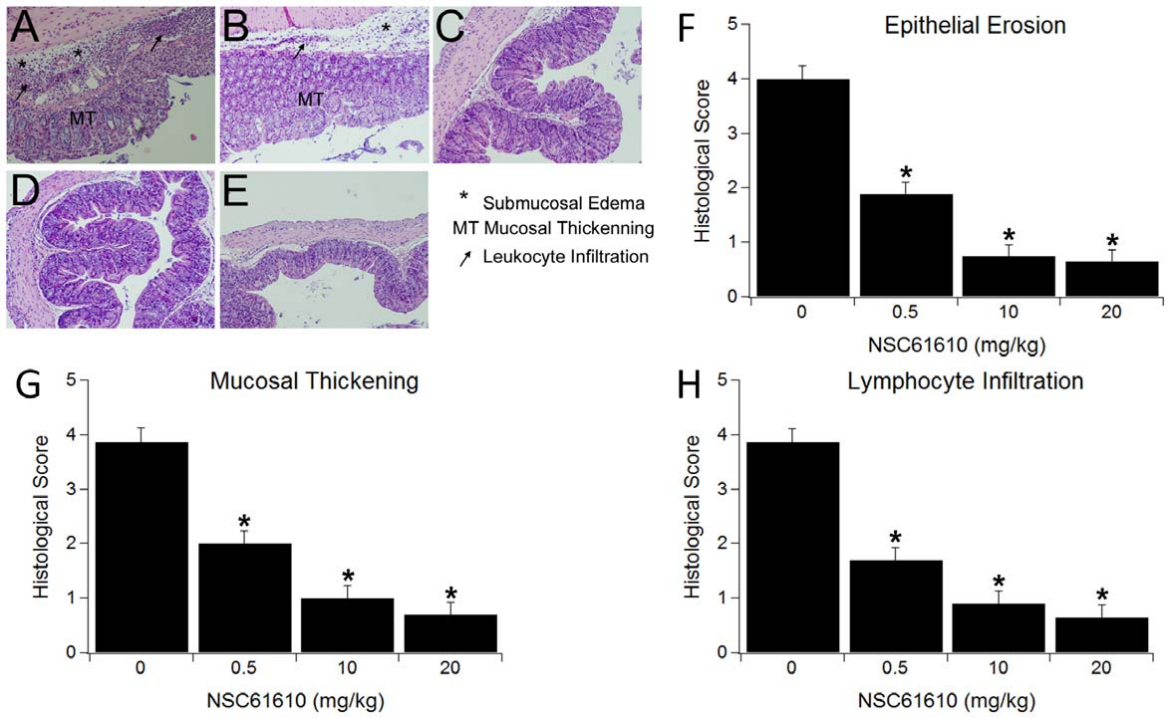
Oral treatment with NSC61610 ameliorates inflammatory lesions in mice with inflammatory bowel disease. Mice were challenged with 2.5% dextran sodium sulfate in the drinking water for 7 days. Representative photomicrographs from the control (A–B) and NSC61610 treatment (C–E) groups are illustrated. Colonic specimens underwent blinded histological examination and were scored based on epithelial erosion (F), mucosal wall thickening (G), and leukocyte infiltration (H). Data are represented as mean ± standard error (n = 10). Bars with an asterisk indicate that a treatment is significantly different from its control (*P<0.05*).

To determine the effect of NSC61610 on colonic inflammation we performed a dose-response study. Specifically, mice received placebo or were treated orally with increasing concentrations of NSC61610 (0.5, 10 and 20 mg/kg body weight) for 7 days during a DSS challenge. After 7 days, mice treated with NSC61610 had a significantly reduced disease activity index (DAI) compared to untreated control mice (Fig. 3A). Based on the gross pathological observation from Figure 3B, 3C, and 3D, NSC61610 treatment significantly decreased inflammation caused by DSS in colon, spleen and MLN. To more closely examine the effect of NSC61610, colonic specimens were examined histologically for the presence of inflammatory lesions. Our data indicate that NSC61610 significantly reduced epithelial erosion, mucosal thickening and leukocyte infiltration in mice with DSS colitis (Fig. 4).

### NSC61610 Modulates Colonic Gene Expression

**Figure 5:**
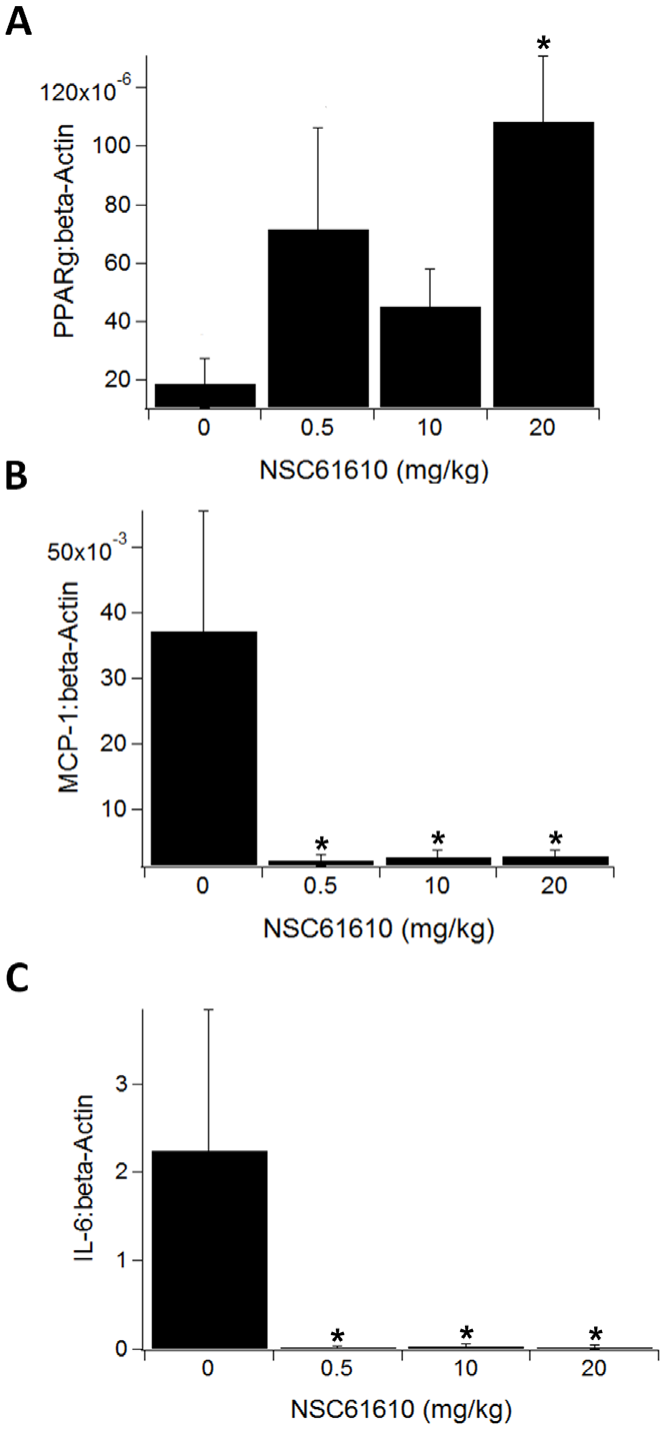
Modulation of colonic gene expression by oral treatment with NSC61610. Mice were challenged with 2.5% dextran sodium sulfate in the drinking water for 7 days. Colonic mRNA expression of peroxisome proliferator-activated receptor γ (PPAR γ) (A), monocyte chemoattractant protein-1 (MCP-1) (B), and interleukin-6 (IL-6) (C) were assessed by quantitative real-time RT-PCR. Data are represented as mean ± standard error (n = 10). Bars with an asterisk indicate that a treatment is significantly different from its control (*P<0.05*).

Our previous research suggested that ABA activates PPAR γ, and PPAR γ agonists have been successfully used in the treatment of IBD [30]. Thus, we sought to determine whether NSC61610 modulates gene expression in a manner that resembled established agonists of PPAR γ such as rosiglitazone or conjugated linoleic acid. Here, we found evidence of PPAR γ-mediated effects in colons of NSC61610-treated mice. For instance, NSC61610 increased the PPAR γ gene expression in colon compared with control mice (Fig. 5A). In addition, NSC61610 significantly lowered expression of inflammatory mediators including monocyte chemoattractant protein-1 (MCP-1) (Fig. 5B) and interleukin-6 (IL-6) (Fig. 5C) in colons of DSS-challenged mice.

### NSC61610 Influences the Phenotype and Distribution of Immune Cells in Mice with IBD

**Figure 6:**
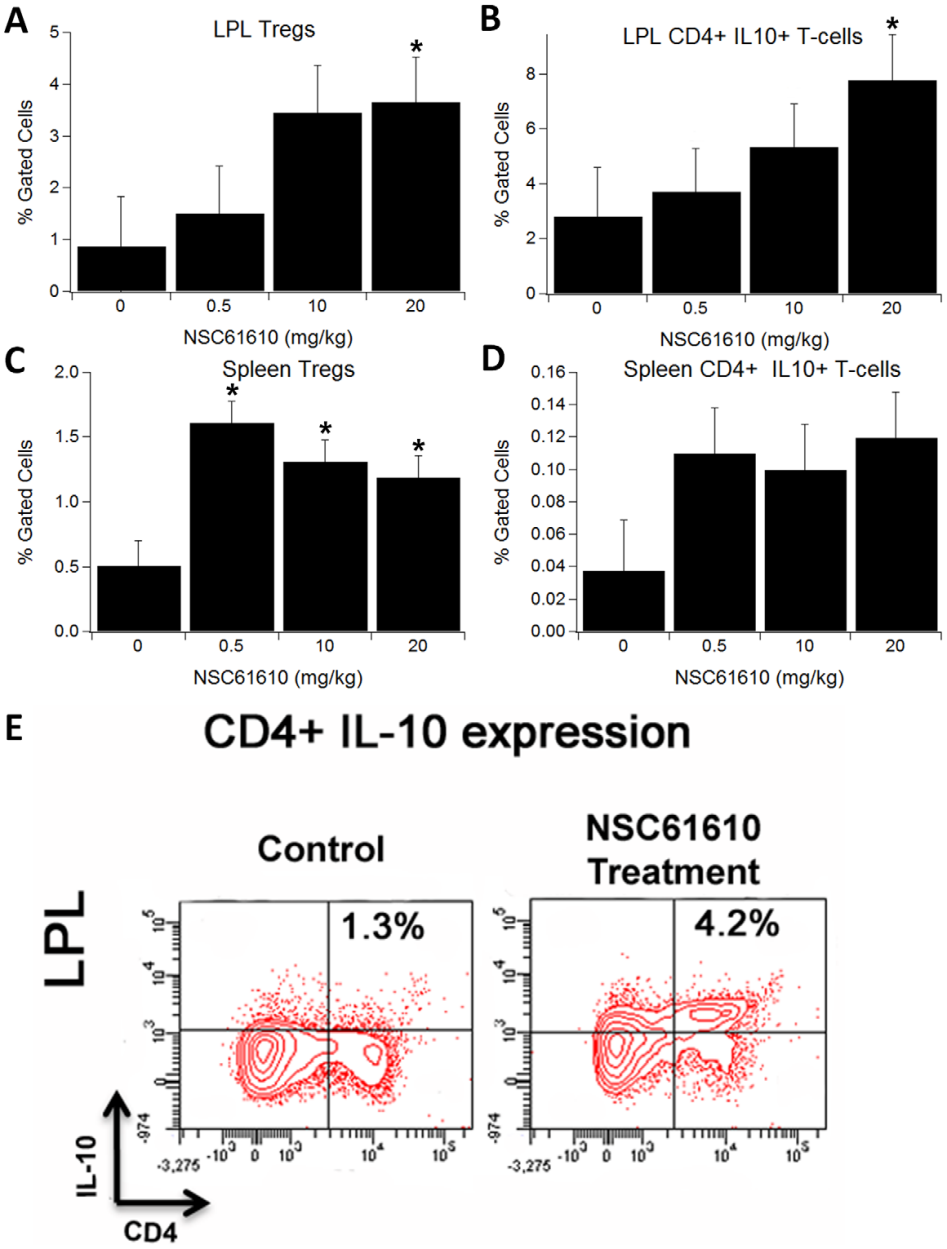
Oral treatment with NSC61610 on the distribution of immune cell subsets in colonic lamina propria and spleen. Colonic lamina propria lymphocytes (LPL, A, B, and E) and spleen (C and D), were immunophenotyped to identify regulatory T cells (Treg) and CD4+IL-10+ T cell subsets through flow cytometry. Data are represented as mean ± standard error (n = 10). Bars with an asterisk indicate that a treatment is significantly different from its control (*P<0.05*).

**Figure 7:**
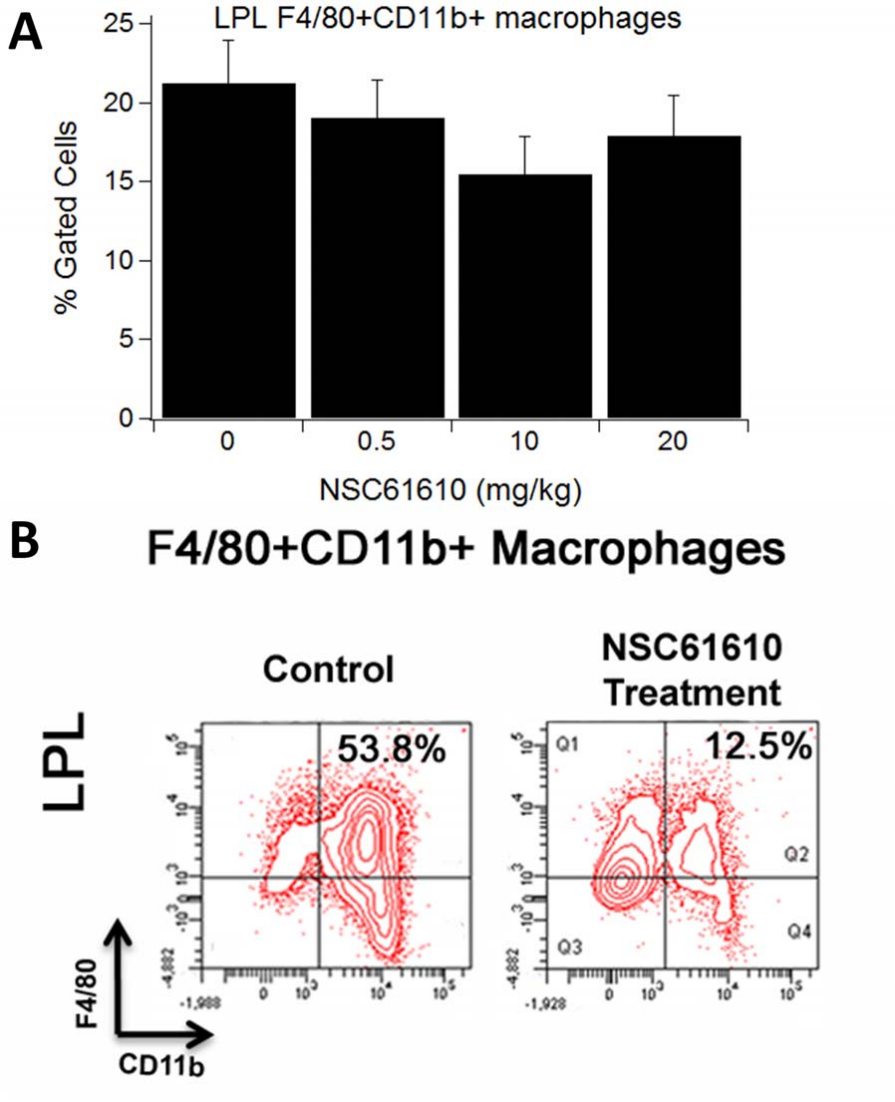
Oral treatment with NSC61610 on the distribution of macrophages in colonic lamina propria. Colonic lamina propria lymphocytes (LPL) were immunophenotyped to identify F4/80+CD11b+ macrophage subsets through flow cytometry. Data are represented as mean ± standard error (n = 10). Bars with an asterisk indicate that a treatment is significantly different from its control (*P<0.05*).

To determine the effect of NSC61610 on immune cell subsets, we performed flow cytometric analysis on cells isolated from the colon, spleen, MLN, and blood. Our analyses indicated that NSC61610 significantly increased the percentage of Treg cells in colon, spleen, and blood (Fig. 6). The highest concentration NSC61610 (20 mg/kg) also significantly increased the percentages of CD4+ IL10+ T cells in colon, spleen, MLN, and blood. In addition, NSC61610 numerically reduced the percentage of F4/80+CD11b+ macrophages infiltrating the colonic lamina propria (Fig. 7).

### NSC61610 Reduces Disease Activity and Inflammatory Lesions via a PPAR γ-dependent Mechanism

**Figure 8:**
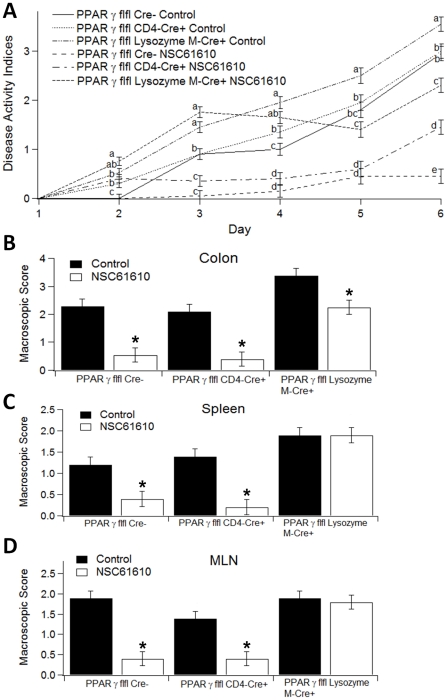
Effect of tissue-specific PPAR γ deletion and oral NSC61610 treatment in experimental inflammatory bowel disease. Mice were challenged with 2.5% dextran sodium sulfate in the drinking water. Panel A illustrates the effect of oral NSC61610 treatment on disease severity. Means within time points with different letterssuperscripts are significant different (*P*<0.05). Panels B–D illustrate the effect of oral NSC61610 on macroscopic lesions in the colon (B), spleen (C), and mesenteric lymph nodes (MLN) (D). Data are represented as mean ± standard error (n = 10). Bars with an asterisk indicate that a treatment is significantly different from its control (*P<0.05*).

To investigate whether the beneficial effects of NSC61610 in IBD required expression of PPAR γ in T cells or macrophages, wild-type (PPAR γ fl/fl, Cre-) mice, macrophage-specific PPAR γ null mice (PPAR γ fl/fl; lysozyme M-Cre+) and T cell-specific PPAR γ null mice (PPAR γ fl/fl; CD4-Cre+) were challenged with 2.5% DSS in the drinking water for 6 days, and disease activity was monitored daily. Macrophage-specific PPAR γ null mice had worsened disease activity throughout the challenge period. From day 4, macrophage-specific PPAR γ null mice had a significantly higher disease activity compared with PPAR γ fl/fl Cre- and PPAR γ fl/fl; CD4-Cre+ mice in both control and treatment groups (Fig. 8A). In line with the DAI scores, both the colons and spleens were significantly more inflamed in PPAR γ fl/fl; Lysozyme M-Cre+ mice than PPAR γ fl/fl Cre- and PPAR γ fl/fl; CD4-Cre+ mice (Fig. 8B and 8C).

Based on the results of the dose response study showing maximum anti-inflammatory efficacy at the highest dose tested, we used an oral dose of 20 mg NSC61610/kg body weight via gastric gavage in this study. NSC61610 treatment significantly reduced DAI compared to untreated control mice following the DSS challenge (Fig. 8A). To more closely examine the effect of NSC61610 on immunopathology caused by DSS, colons, spleens and MLNs were examined macroscopically for the presence of inflammatory lesions. Our data indicate that NSC61610 significantly reduced macroscopic inflammatory lesions in PPAR γ-expressing and T cell-specific PPAR γ null mice with DSS colitis (Fig. 8B, 8C and 8D). However, the therapeutic effect of NSC61610 on IBD was abrogated in spleens and MLNs of macrophage-specific PPAR γ null mice (Fig. 8C and 8D). Thus, we posit that the anti-inflammatory efficacy of NSC61610 is dependent on PPAR γ expression in macrophages.

## Discussion

LANCL2 has received some recent attention as a potential therapeutic target due to its function related to ABA binding and signaling [6] and the recent discovery of an alternative membrane-based mechanism of PPAR γ activation [8]. Furthermore, we determined the LANCL2 expression in a series of mouse tissues, which showed that beside brain and testis, LANCL2 is also expressed in other tissues, such as thymus, spleen, colon, and PP, which indicates the possible relationship between LANCL2 and immune responses and suggest the broader potential of LANCL2 as a therapeutic target.

ABA has been shown to play an important role in regulating immune and inflammatory processes [8] via PPAR γ-mediated responses in mouse models of obesity-related inflammation, diabetes, atherosclerosis, and IBD [30], [31], [32], [33], [34]. We discovered that ABA activates PPAR γ and the loss of PPAR γin immune cells impairs its ability to normalize blood glucose concentrations and ameliorate macrophage infiltration in the white adipose tissues of obese mice [32]. PPAR γ suppresses the expression of pro-inflammatory cytokines and chemokines by antagonizing the activities of transcription factors, enhancing nucleocytoplasmic shuttling of the activated p65 subunit of NF-κB, and targeting co-repressor complexes onto inflammatory gene promoters [8]. These molecular changes induced by PPAR γ agonists are linked to anti-inflammatory efficacy in mouse models of systemic and mucosal inflammation [8], [31], [35].

At the molecular level, the mechanism underlying ABA-mediated activation of PPAR γ is independent of direct binding to PPAR γ's LBD [8]. The inability of ABA to bind directly to the LBD was further validated by competitive ligand-binding assays demonstrating the inability of ABA to displace the trace for binding to the PPAR γ LBD. These results suggested existence of a potential molecular target for ABA upstream of PPAR γ. ABA was shown to increase cAMP accumulation in insulin-secreting pancreatic β-cell lines, splenocytes, and macrophages [34], [36]. LANCL2 represents a possible membrane-associated target for ABA involved in the initiation of the cAMP signal. In a previous study on LANCL2 *in silico*, we used homology modeling approaches to construct a three dimensional structure of LANCL2 and identified a putative ABA-binding site on the surface of LANCL2 [10]. A series of *in vitro* experiments further demonstrated that LANCL2 is a putative novel target for the discovery and development of orally active, broad-based drugs against inflammatory, infectious and chronic metabolic diseases. In this study, we used LANCL2 docking studies for the large-scale, high-throughput screening of thematic compound databases for novel drugs for treating inflammatory, infectious and chronic diseases. These molecular modeling studies facilitated that discovery of numerous potential ligands of LANCL2 from NCI Diversity Set II, ChemBridge, ZINC natural products, FDA-approved drugs database and ABA analogs. Furthermore, in order to validate these predictions and determine the anti-inflammatory efficacy of the top ranked compound, denoted as NSC61610 in NCI Diversity Set II, a series of *in vitro* and pre-clinical efficacy studies using a mouse model of DSS-induced colitis were performed.

Previously, we have reported that ABA transactivates PPAR γ *in vitro* and suppresses systemic inflammation similar to other PPAR γ agonists. Since both ABA and NSC61610 target LANCL2, NSC61610 might also act via PPAR γ activation.Experimental results show that NSC61610 treatment activates PPAR γ in raw macrophages, thereby providing evidence of a potential signaling relationship between LANCL2 and PPAR γ and indicating that NSC61610 might target the LANCL2-PPAR γ axis *in vitro*. To investigate the importance of LANCL2 in NSC61610-mediated activation of PPAR γ, we determined whether knocking down LANCL2 in raw macrophages by using siRNA impaired or abrogated the effect of NSC61610 on PPAR γ reporter activity. Our findings indicate that knocking down LANCL2 significantly attenuates the effect of NSC61610 on PPAR γ activity. These findings are consistent with the prediction of our previous studies that ABA activates PPAR γ reporter activity in an LANCL2-dependent manner [8]. In addition, to determine whether NSC61610-induced activation of PPAR γ is dependent on adenylate cyclase-cAMP signaling, we measured the NSC61610-induced PPAR gamma activity with or without 2′5′-dideoxyadenosine, AC-specific inhibitor. AC transmits signals by converting adenosine triphosphate to cAMP, a second messenger. The results show addition of the AC-specific inhibitor prevented NSC61610-induced PPAR gamma activation, which indicates NSC61610-induced PPAR gamma activation is AC/cAMP dependent. We also examined whether the AC-specific inhibitor influenced PPAR γ activity in cells with knockdown of LANCL2. The PPAR γ activity was not further inhibited with addition of AC-specific inhibitor that indicates that LANCL2 is one receptor upstream of AC-cAMP signaling pathway, which is in line with our previous findings that LACNCL2 stimulation is followed by cAMP accumulation [8].

Herein, we demonstrate for the first time that oral NSC61610 treatment significantly ameliorates colonic inflammation and clinical activity in mice with experimental IBD. Consistent with *in vitro* results in raw macrophages showing increased PPAR γ reporter activity, we found that NSC61610 treatment upregulated colonic PPAR γ gene expression in mice with IBD. In addition, NSC61610 significantly decreased inflammatory mediators in the colonic mucosa, including MCP-1 and IL-6. MCP-1 plays an important role in the pathogenesis of colitis in relation to the recruitment of immune cells, and the absence of this chemokine is associated with a significant reduction in inflammation [37]. CD4+ T cells at the site of inflammation are critically dependent on anti-apoptotic IL-6 signaling. This circle of T cell accumulation, mediated by apoptosis resistance, which leads to chronic inflammation, can be blocked by anti-IL-6 receptor antibodies [38]. In combination with transforming growth factor-β, IL-6 is also involved in differentiation of naïve CD4+ T cells into a pro-inflammatory T helper (Th) 17 phenotype that has been associated with autoimmunity [39].

At the cellular level, we observed that oral NSC61610 treatment significantly increased the percentages of Treg cells in the colon, spleen, and blood of mice. Tregs are important for the maintenance of intestinal self-tolerance and suppression of inflammation. Therapies that increase Treg numbers and function are under intense investigation and may prove to be promising treatments for patients with IBD [40], [41]. In addition, treatment with NSC61610 at the highest concentration also significantly increased the percentages of CD4+IL10+ T cells in colon, spleen, MLN, and blood. IL-10 is a regulatory cytokine that inhibits both antigen presentation and subsequent pro-inflammatory cytokine release, and it is proposed as a potent anti-inflammatory biological therapy in chronic IBD [42], [43]. Furthermore, NSC61610 reduced the percentage of infiltrating F4/80+CD11b+ macrophages in the colonic lamina propria, a possible source of inflammatory mediators. Our previous research indicated that ABA ameliorates experimental IBD by suppressing immune cell infiltration [31], [35], which meshes with the immunosuppressive responses seen with NSC61610. This commonality may indicate that ABA and NSC61610 do indeed share similar anti-inflammatory mechanisms that are possibly initiated via LANCL2 binding. In previous study on ABA, we found T cell PPAR γ is a crucial mediator of ABA's anti-inflammatory responses. The absence of PPAR γ in T cells essentially abolished the ability of ABA to decrease experimental IBD, including the anti-inflammatory action of ABA in the colon. T cell PPAR γ was required for the regulation of Treg cell number in the mucosal inductive (MLNs) and effector (colonic lamina propria) sites [30]. To investigate the cell specificity and molecular targets underlying the anti-inflammatory mechanism of NSC61610 *in vivo*, we tested whether the beneficial effect of NSC61610 in IBD required expression of PPAR γ in T cells or macrophages. Since the beneficial effect of NSC61610 treatment on IBD was abrogated in macrophage-specific PPAR γ null mice, we posit that the anti-inflammatory efficacy of NSC61610 is dependent on PPAR γ expression in macrophages. Although the anti-inflammatory mechanisms of ABA and NSC61610 are involve LANCL2, their differences in cell specificity indicate that activation of LANCL2 possibly can trigger multiple signal pathways in different cells which would finally promote PPAR γ expression and exert anti-inflammatory efficacy. Based on this finding, synergistic effect of ABA and NSC61610 will be tested in our future studies.

In an attempt to identify additional targets to shed new light on potential alternative mechanisms of action for NSC61610 we used TarFisDock to analyze the reverse docking results of this compound. Putative targets were selected by ranking the values of the interaction energy, which consist of van der Waals and electrostatic interaction terms. The top 10 reverse docking results of NSC61610 are shown in Table S6. Of note, we show novel data indicating that NSC61610 may decrease inflammation by alternatively targeting the leukotriene A4 hydrolase, which is an enzyme linked to the production of inflammatory lipid mediators such as leukotrienes, suggesting a possible role of NSC61610 in modulating the synthesis of lipid mediators such as prostaglandins and leukotrienes that worsen the pathogenesis of IBD. It is obvious that the docking energy scores obtained in TarFisDock are more negative than those in AutoDock and AutoDock Vina, which is generated by the differences between scoring functions. TarFisDock was developed on the basis of the DOCK (version 4.0) program with a force-field-based scoring function, while AutoDock and AutoDock Vina use an empirical scoring function. Different scoring functions have been compared and assessed in previous studies [44]. In terms of the TarFisDock energy score, this value represented strength of ligand association where a more negative value translated to a stronger predicted protein-ligand complex.

In conclusion, this study employed an integrated drug discovery pipeline consisting of molecular modeling approaches followed by experimental validation. We performed large-scale screening of compound libraries based on predicted binding to an LANCL2 binding site and identified novel putative compounds for the treatment of inflammatory diseases. NSC61610, the top ranked lead compound based on binding free energy, significantly ameliorated experimental IBD in mice in a LANCL2- and PPAR γ-dependent manner. These results confirm that LANCL2 is a novel therapeutic target for inflammatory diseases and NSC61610 is a potential new drug.

## Supporting Information

Table S1 **Oligonucleotide sequences for quantitative real-time PCR.**
(DOCX)

Table S2 **Docking results of compounds in NCI Diversity Set II to lanthionine synthetase C-like 2, ranked by the lowest binding energy (N = 1,364 compounds).**
(DOCX)

Table S3 **Docking results of compounds in ChemBridge to lanthionine synthetase C-like 2, ranked by the lowest binding energy (N = 884,105 compounds).**
(DOCX)

Table S4 **Docking results of compounds in ZINC Natural Products database to lanthionine synthetase C-like 2, ranked by the lowest binding energy (N = 89,425 compounds).**
(DOCX)

Table S5 **Docking results of compounds in Food and Drug Administration-approved drugs database to lanthionine synthetase C-like 2, ranked by the lowest binding energy (N = 3,180 compounds).**
(DOCX)

Table S6 **Potential therapeutic targets of NSC61610.**
(DOCX)
