# Supplementary material for: Computational Modeling-Based Discovery of Novel Classes of Anti-Inflammatory Drugs That Target Lanthionine Synthetase C-Like Protein 2
Source: PLoS One. 2012 Apr 11;7(4):e34643. doi: 10.1371/journal.pone.0034643 (PMC3324509; doi:10.1371/journal.pone.0034643)
Supplement: Table S1 — Oligonucleotide sequences for quantitative real-time PCR. (DOCX) [file pone.0034643.s001.docx]

Supplementary Table S1. Oligonucleotide sequences for quantitative real-time PCR. ^a,b^

| Primer | Sequence | Length | Accession Number |
| --- | --- | --- | --- |
| β-actin F | 5’ CCCAGGCATTGCTGACAGG3’ | 141 | X03672 |
| β-actin R | 5’ TGGAAGGTGGACAGTGAGGC3’ |  |  |
| PPAR γ F | 5’ AGAACCTGCATCTCCACCTT3’ | 117 | NM_011146 |
| PPAR γ R | 5’ ACAGACTCGGCACTCAATGG3’ |  |  |
| IL-6 F | 5’ TTTCCTCTGGTCTTCTGGAG3’ | 92 | NM_031168 |
| IL-6 R | 5’ CTGAAGGACTCTGGCTTTGT3’ |  |  |
| MCP-1 F | 5’ CTTTGAATGTGAAGTTGACCC3’ | 129 | NM_011333 |
| MCP-1 R | 5’ AGGCATCACAGTCCGAGTC3’ |  |  |
| TNF-α F | 5’ AGGCATCACAGTCCGAGTC3’ | 137 | NM_013693 |
| TNF-α R | 5’ AGGCATCACAGTCCGAGTC3’ |  |  |

^a^ F, forward; R, reverse. PCR primer pairs were designed for an optimal annealing temperature of 57.2°C and product lengths between 92 and 141 base pairs.

^b^ When plotting threshold cycle versus log starting quantity (pg), standard curves had slopes between -1.932 and -2.989; PCR efficiencies between 105.3 and 229.3 and R^2^ above 0.98 mostly.
