# Supplementary material for: Computational Modeling-Based Discovery of Novel Classes of Anti-Inflammatory Drugs That Target Lanthionine Synthetase C-Like Protein 2
Source: PLoS One. 2012 Apr 11;7(4):e34643. doi: 10.1371/journal.pone.0034643 (PMC3324509; doi:10.1371/journal.pone.0034643)
Supplement: Table S2 — Docking results of compounds in NCI Diversity Set II to lanthionine synthetase C-like 2, ranked by the lowest binding energy (N = 1,364 compounds). (DOCX) [file pone.0034643.s002.docx]

Supplementary Table S2. Docking results of compounds in NCI Diversity Set II to lanthionine synthetase C-like 2, ranked by the lowest binding energy (N=1,364 compounds).

| **ZINC Number** | **Name** | **Chemical Structure** | **Lowest**  **Binding**  **Energy**  **(**kcal/mol**)** |
| --- | --- | --- | --- |
| ZINC01690699  (NSC61610) | 1-N,4-N-bis[3-(1H-benzimidazol-2-yl)phenyl]benzene-1,4-dicarboxamide | 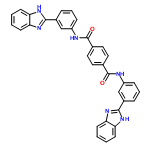 | -11.1 |
| ZINC29589888 | 2-[2-[(6-oxo-5H-phenanthridin-3-yl)carbamoyl]phenyl]benzoic acid | 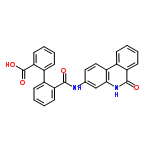 | -10.5 |
| ZINC13130018 | 6-(1,3-dihydrophenanthro[9,10-d]imidazol-2-ylidene)cyclohexa-2, 4-dien-1-one | 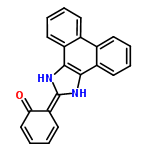 | -10.3 |
| ZINC01726776 | 3-(4-chloro-6-phenoxy-1,3,5-triazin-2-yl)-1-phenylindole | 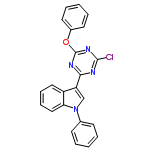 | -10.2 |
| ZINC01736228 | (2R)-5-phenyl-2-[(2R)-5-phenyl-2,3-dihydro-1,3-benzoxazol-2-yl]-2, 3-dihydro-1,3-benzoxazole | 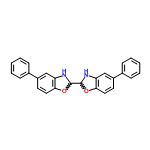 | -10.2 |
| ZINC04783229 | 1-N,4-N-bis(3-phenylphenyl)piperazine-1,4-dicarboxamide | 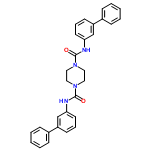 | -10.1 |
| ZINC00990239 | 3-(4,5-dimethylbenzo[h][1, 6]naphthyridin-1-ium-2-yl)-2-methylquinolin-4-amine | 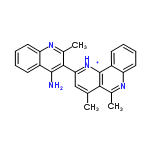 | -10 |
| ZINC18057104 | 4-[(1-methyl-6-nitroquinolin-1-ium-4-yl)amino]-N-[4-[(1-methylpyridin-1- ium-4-yl)amino]phenyl]benzamide | 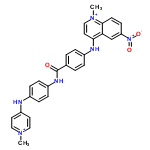 | -10 |
| ZINC04214344 | Genostrychnine | 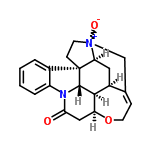 | -9.7 |
| ZINC04720972 | 2-hydroxy-N-(4-methoxyphenyl)-11H-benzo[a]carbazole-3-carboxamide | 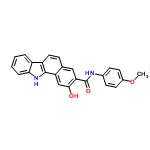 | -9.6 |
