# Supplementary material for: Computational Modeling-Based Discovery of Novel Classes of Anti-Inflammatory Drugs That Target Lanthionine Synthetase C-Like Protein 2
Source: PLoS One. 2012 Apr 11;7(4):e34643. doi: 10.1371/journal.pone.0034643 (PMC3324509; doi:10.1371/journal.pone.0034643)
Supplement: Table S3 — Docking results of compounds in ChemBridge to lanthionine synthetase C-like 2, ranked by the lowest binding energy (N = 884,105 compounds). (DOCX) [file pone.0034643.s003.docx]

Supplementary Table S3. Docking results of compounds in ChemBridge to lanthionine synthetase C-like 2, ranked by the lowest binding energy (N=884,105 compounds).

| **ZINC Number** | **Name** | **Chemical Structure** | **Lowest**  **Binding**  **Energy**  **(**kcal/mol**)** |
| --- | --- | --- | --- |
| ZINC22146248 | 3-[4-(5,5-dioxidodibenzo[b,d]thien-2-yl)-5-phenyl-1H-imidazol-2-yl]-3a,7a-dihydro-1H-indole | 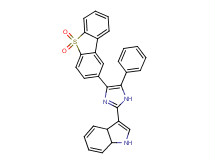 | -10.9 |
| ZINC02848490 | N-(6-chloro-1,3-dioxo-1H-benzo[de]isoquinolin-2(3H)-yl)-9-hydroxy-9H-fluorene-9-carboxamide | 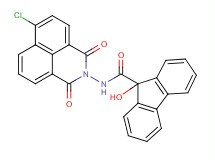 | -10.8 |
| ZINC13081141 | 2-[2-(4-hydroxyphenyl)-5-phenyl-1H-imidazol-4-yl]-7-nitro-9H-fluoren-9-one | 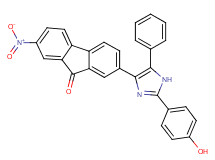 | -10.8 |
| ZINC05799242 | 2-(1,3-benzodioxol-5-yl)-4,4-dimethyl-6-phenyl-1,4,5,6-tetrahydroimidazo[4,5-e]indazole | 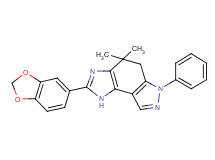 | -10.5 |
| ZINC04602469 | 2-[4-amino-6-(dimethylamino)-1,3,5-triazin-2-yl]-3-dibenzo[b,d]furan-2-ylacrylonitrile | 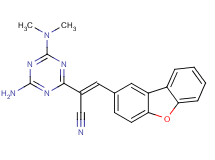 | -10.3 |
| ZINC02909739 | 1-phenyl-5-{[(5-phenyl-1,3,4-thiadiazol-2-yl)amino]methylene}-2,4,6(1H,3H,5H)-pyrimidinetrione | 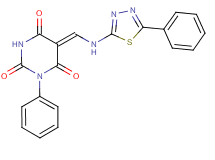 | -10.2 |
| ZINC14740873 | 7-[(6-chloro-2H-chromen-3-yl)methyl]-3-(3,4-dihydro-2H-chromen-3-yl)-1-methyl-5,6,7,8-tetrahydroimidazo[1,5-a]pyrazine | 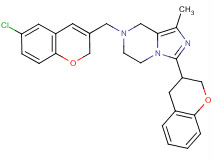 | -10.2 |
| ZINC05338533 | 1-phenyl-4,11-dihydroimidazo[4,5-e]naphtho[2,3-b][1,4]diazepin-2(1H)-one | 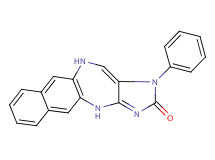 | -10.2 |
| ZINC08387449 | 1-phenyl-4,11-dihydroimidazo[4,5-e]naphtho[2,3-b][1,4]diazepin-2(1H)-one | 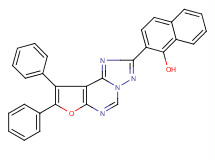 | -10.2 |
| ZINC05564677 | ethyl 4-[5-(4-fluorophenyl)-2-furyl]-2-oxo-6-phenyl-1,2-dihydro-5-pyrimidinecarboxylate | 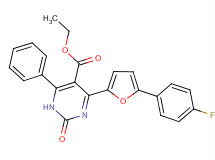 | -10.1 |
