# Supplementary material for: Computational Modeling-Based Discovery of Novel Classes of Anti-Inflammatory Drugs That Target Lanthionine Synthetase C-Like Protein 2
Source: PLoS One. 2012 Apr 11;7(4):e34643. doi: 10.1371/journal.pone.0034643 (PMC3324509; doi:10.1371/journal.pone.0034643)
Supplement: Table S4 — Docking results of compounds in ZINC Natural Products database to lanthionine synthetase C-like 2, ranked by the lowest binding energy (N = 89,425 compounds). (DOCX) [file pone.0034643.s004.docx]

Supplementary Table S4. Docking results of compounds in ZINC Natural Products database to lanthionine synthetase C-like 2, ranked by the lowest binding energy (N=89,425 compounds).

| **ZINC Number** | **Name** | **Chemical Structure** | **Lowest**  **Binding**  **Energy**  **(**kcal/mol**)** |
| --- | --- | --- | --- |
| ZINC03845566 | 3,7-bis(2-oxo-1H-indol-3-ylidene)-1,5-dihydropyrrolo[2,3-f]indole-2, 6-dione | 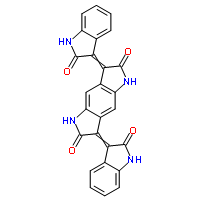 | -12.8 |
| ZINC03848528 | 1-amino-3-[(4-amino-9,10-dioxoanthracen-2-yl)amino]anthracene-9,10-dione | 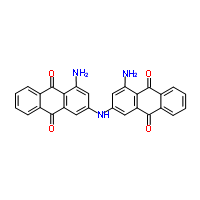 | -12.0 |
| ZINC05220992 | benzo[lmn]diquinazolino[2,1-b:2',3'-i][3,8]phenanthroline-5,9,11,19-tetrone | 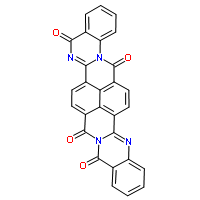 | -11.7 |
| ZINC08792261 | N-1,3-benzothiazol-2-yl-2-[(9-oxo-9H-benzo[c]indolo[3,2,1-ij][1,5]naphthyridin-5-yl)oxy]propanamide | 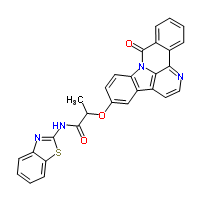 | -11.5 |
| ZINC09033168 | 1-(2-dibenzofuran-3-ylhydrazinyl)-[1]benzofuro[3,2-e]indol-2-one | 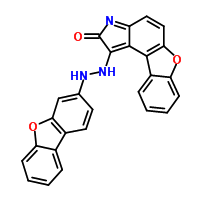 | -11.4 |
| ZINC02121309 | 2-(2-dibenzofuran-2-ylhydrazinyl)-[1]benzofuro[2,3-f]indol-1-one | 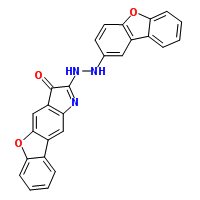 | -10.8 |
| ZINC12654409 | 3',11'-Dihydroxy-3H-spiro[2-benzofuran-1,7'-dibenzo[c,h]xanthen]-3-one | 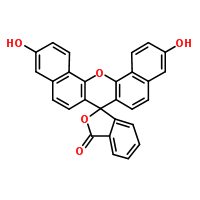 | -10.7 |
| ZINC03843486 | [1,4]benzodioxino[2,3-b][1,4]benzodioxino[2',3':5,6]pyrazino[2,3-g]quinoxaline | 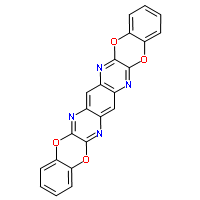 | -10.6 |
| ZINC04701574 | 6-chloro-3-[(2E)-2-[1-(2-oxochromen-3-yl)ethylidene]hydrazinyl]indol-2- one | 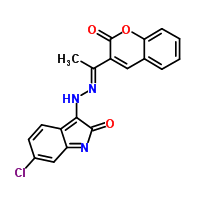 | -10.6 |
| ZINC04266071 | (2Z)-2-(3-oxo-1H-indol-2-ylidene)naphtho[3,2-e][1]benzothiole-1,6, 11-trione | 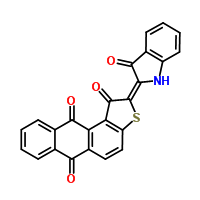 | -10.4 |
