# Supplementary material for: Computational Modeling-Based Discovery of Novel Classes of Anti-Inflammatory Drugs That Target Lanthionine Synthetase C-Like Protein 2
Source: PLoS One. 2012 Apr 11;7(4):e34643. doi: 10.1371/journal.pone.0034643 (PMC3324509; doi:10.1371/journal.pone.0034643)
Supplement: Table S5 — Docking results of compounds in Food and Drug Administration-approved drugs database to lanthionine synthetase C-like 2, ranked by the lowest binding energy (N = 3,180 compounds). (DOCX) [file pone.0034643.s005.docx]

Supplementary Table S5. Docking results of compounds in Food and Drug Administration-approved drugs database to lanthionine synthetase C-like 2, ranked by the lowest binding energy (N=3,180 compounds).

| **ZINC Number** | **Name** | **Chemical Structure** | **Function** | **Lowest**  **Binding**  **Energy**  **(**kcal/mol**)** |
| --- | --- | --- | --- | --- |
| ZINC03830554 | 4-amino-3-[[4-[4-[(1-amino-4-sulfonatonaphthalen-2-yl)diazenyl]phenyl] phenyl]diazenyl]naphthalene-1-sulfonate | 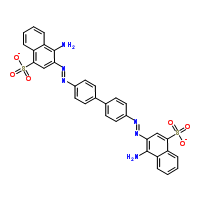 | inhibit amyloid polymerization | -10.5 |
| ZINC11678081 | Carminomycin | 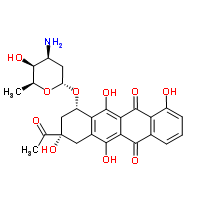 | antibiotics, antineoplastic | -9.9 |
| ZINC08552616 | Algestone Acetophenide | 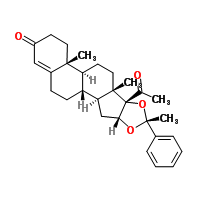 | progestins, contraceptives,anti-inflammatary agents | -9.7 |
| ZINC08101049 | Acetyldigitoxins | 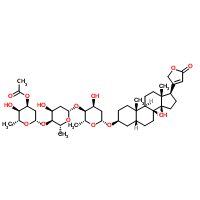 | anti-arrhythmia, cardiotonic agents | -9.5 |
| ZINC08101053 | Aclacur | 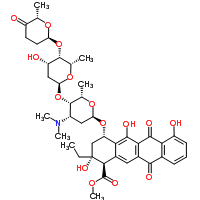 | antibiotics, antineoplastic | -9.4 |
| ZINC08101078 | Digitoxin | 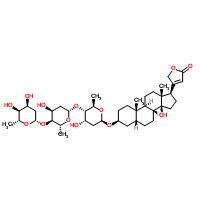 | anti-arrhythmia, cardiotonic agents | -9.4 |
| ZINC01529463 | Estrone hydrogen sulfate | 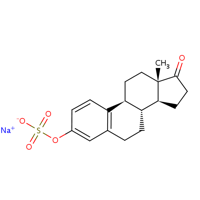 | female hormone | -9.4 |
| ZINC03830332 | 4,4'-((2,4-Dihydroxy-5-(hydroxymethyl)-1,3-phenylene)bis(azo))bisnaphthalene-1-sulphonic acid | 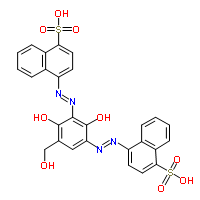 |  | -9.4 |
| ZINC11592963 | Idarubicin | 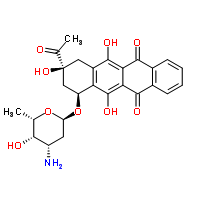 | antibiotics, antineoplastic | -9.4 |
| ZINC03830975 | Itraconazole | 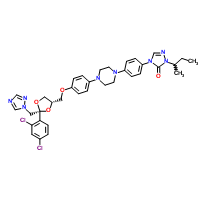 | antifungal agents | -9.3 |
