# Supplementary material for: Computational Modeling-Based Discovery of Novel Classes of Anti-Inflammatory Drugs That Target Lanthionine Synthetase C-Like Protein 2
Source: PLoS One. 2012 Apr 11;7(4):e34643. doi: 10.1371/journal.pone.0034643 (PMC3324509; doi:10.1371/journal.pone.0034643)
Supplement: Table S6 — Potential therapeutic targets of NSC61610. (DOCX) [file pone.0034643.s006.docx]

Supplementary Table S6. Potential therapeutic targets of NSC61610.

| **PDB_ID** | **Energy Score** | **Target Name** | **Processes and Diseases** |
| --- | --- | --- | --- |
| 1Q0N | -63.44 | 6-Hydroxymethyl-7, 8-dihydropterin pyrophosphokinase (HPPK) | Infections;  Microbial infections |
| 1HS6 | -61.89 | Leukotriene A4 hydrolase | Inflammation;  leukotriene synthesis;  Esophageal cancer |
| 1K6W | -61.24 | Cytosine deaminase | Epigenetic events;  Cancer |
| 1HDT | -59.59 | Serine Proteinase alpha-thrombin | Haemostatic Disorders |
| 1FNO | -58.78 | Peptidase |  |
| 1LGR | -57.52 | Glutamine Synthetase | Alzheimer's Disease,  Huntington Disease |
| 1HDT | -57.5 | Serine Proteinase alpha-thrombin | Haemostatic Disorders |
| 1XID | -57.47 | D-Xylose Isomerase | Carbohydrate metabolism |
| 1ED5 | -56.78 | Nitric Oxide Synthase | Vasodilation;  Inflammation |
| 1GPN | -55.86 | Acetylcholinesterase | Alzheimer's Disease,  Cognitive Deficits,  Hypoxic-ischemic  Encephalopathy,  Motor Neurone Disease,  Parkinson's Disease |
